# *Vital Signs:* Prevalence of Multiple Forms of Violence and Increased Health Risk Behaviors and Conditions Among Youths — United States, 2019

**DOI:** 10.15585/mmwr.mm7005a4

**Published:** 2021-02-05

**Authors:** Corinne David-Ferdon, Heather B. Clayton, Linda L. Dahlberg, Thomas R. Simon, Kristin M. Holland, Nancy Brener, Jennifer L. Matjasko, Ashley S. D’Inverno, Leah Robin, Derrick Gervin

**Affiliations:** ^1^Division of Violence Prevention, National Center for Injury Prevention and Control, CDC; ^2^Division of Overdose Prevention, National Center for Injury Prevention and Control, CDC; ^3^Division of Adolescent and School Health, National Center for HIV/AIDS, Viral Hepatitis, STD, and TB Prevention, CDC.

## Abstract

**Introduction:**

Experiencing violence, especially multiple types of violence, can have a negative impact on youths’ development. These experiences increase the risk for future violence and other health problems associated with the leading causes of morbidity and mortality among adolescents and adults.

**Methods:**

Data from the 2019 national Youth Risk Behavior Survey were used to determine the prevalence of high school students’ self-reported experiences with physical fighting, being threatened with a weapon, physical dating violence, sexual violence, and bullying. Logistic regression models adjusting for sex, grade, and race/ethnicity were used to test the strength of associations between experiencing multiple forms of violence and 16 self-reported health risk behaviors and conditions.

**Results:**

Approximately one half of students (44.3%) experienced at least one type of violence; more than one in seven (15.6%) experienced two or more types during the preceding 12 months. Experiencing multiple types of violence was significantly more prevalent among females than among males and among students identifying as gay, lesbian, or bisexual or not sure of their sexual identity than among heterosexual students. Experiencing violence was significantly associated with higher prevalence of all examined health risks and conditions. Relative to youths with no violence experiences, adjusted health risk and condition prevalence estimates were up to seven times higher among those experiencing two types of violence and up to 21 times higher among those experiencing three or more types of violence.

**Conclusions and implications for public health practice:**

Many youths experience multiple types of violence, with potentially lifelong health impacts. Violence is preventable using proven approaches that address individual, family, and environmental risks. Prioritizing violence prevention is strategic to promoting adolescent and adult health.

## Introduction

Violence experienced by high school-aged youths is a significant public health problem. Homicide is the third leading cause of death among persons aged 14–18 years in the United States (*1*). Every day, approximately 360 youths are treated in emergency departments for nonfatal assault-related injuries (*1*). Youths also report experiencing a high prevalence of different types of violence (e.g., fights, dating violence, and bullying) (*2*). Because many types of violence share the same risk factors and experiencing one type of violence increases the risk for experiencing another type of violence, some youths have multiple violence experiences during childhood and adolescence (*3*,*4*).

Adverse childhood experiences, including experiences of violence, are traumatic and can have a negative impact on the brain’s chemistry and physical development related to attention, decision-making, learning, and emotional regulation (*5*,*6*). Adolescence is a critical period for the development of cognitive, emotional, and interpersonal skills, and exposure to violence in the home and community during this period can disrupt healthy brain and associated skill growth (*7*,*8*). These impacts could impair problem-solving, ability to cope with stress, and academic performance. Exposure to violence, especially multiple types of violence, could exacerbate these disruptions in development, which could have a negative impact on health across the life course. Surveys of adults demonstrate that adverse experiences before age 18 years, including violence experiences (e.g., child abuse and neglect and witnessing intimate partner violence), significantly increase the risk for adult chronic health conditions and risk behaviors (e.g., overweight or obesity, smoking, and heavy drinking), depression, and negative socioeconomic outcomes, especially as these adverse experiences accumulate (*9*).

Preventing violence during childhood and adolescence might reduce morbidity and mortality in adolescence and adulthood and improve economic and social outcomes (*10*). Previous research has focused primarily on the health consequences of adverse childhood experiences within the home and violence perpetrated by adults; however, less is known about the potential health effects of adolescents experiencing multiple types of violence in school and community settings (*10*). Addressing this gap could inform the collaborative work of youth-serving partners (e.g., health, education, and justice) to prevent adolescent health risk behaviors and conditions linked to morbidity and premature mortality.

## Methods

This report includes results from CDC’s 2019 Youth Risk Behavior Survey (YRBS). YRBS is a nationally representative, biennial, cross-sectional, complex school-based survey that measures prevalence of health-related behaviors among students in grades 9–12 who attend public and private schools in the United States. The school response rate for the 2019 national YRBS was 75.1%, and the student response rate was 80.3% (*2*). The overall sample size was 13,677 students. Participants answered eight questions related to four types of violence (physical fighting or threatened with a weapon, physical dating violence, sexual violence, and bullying) experienced during the 12 months before the survey (Supplementary Table, https://stacks.cdc.gov/view/cdc/101069). In some schools, students were not asked all the physical fighting and sexual violence questions. The analytic sample was restricted to 9,080 students for whom data were available to assess the presence of all four types of violence experiences. Students were classified into one of four categories based on the number of violence types experienced: zero, one, two, or three or more. The prevalence of students experiencing one, two, and three or more types of violence was examined by sex, race/ethnicity (non-Hispanic white, non-Hispanic black, and Hispanic), grade in school (9–12), and sexual identity (heterosexual, gay/lesbian/bisexual, and not sure) using a test for trend.

Participants also answered questions about 16 health risk behaviors and conditions during the 30 days, 3 months, or 12 months before the survey (Supplementary Table, https://stacks.cdc.gov/view/cdc/101069). Prevalence for each of these health risks was estimated by number of types of violence experiences. Pairwise comparisons were conducted using t-tests and considered significant if p<0.05. Logistic regression models with predicted marginals were used to quantify the associations between violence experiences (the exposure of interest) and each health risk (outcomes of interest), adjusting for sex, race/ethnicity, grade, and sexual identity. Associations are presented as adjusted prevalence ratios (aPRs). Analyses were conducted using SAS callable SUDAAN (version 11.0.3; RTI International).

## Results

Among high school students, 28.7% experienced one type of violence during the 12 months before the survey, 10.8% experienced two types, and 4.8% experienced three or more types (Table 1). This equates to 44.3% students experiencing at least one type and 15.6% two or more types. Sex and sexual identity were significantly associated with experiencing violence. Female students (6.1%) were significantly more likely than were male students (3.4%) to experience three or more types of violence. Students identifying as gay, lesbian, or bisexual (9.6%) or not sure of their sexual identity (7.4%) were significantly more likely than were heterosexual students (3.8%) to report three or more types of violence experiences.

**TABLE 1:** Number of types of violence experiences among high school students, by demographic characteristics — Youth Risk Behavior Survey, United States, 2019

Characteristic	No. of types of violence experiences* % (95% CI)				P-value^†^
	0	1	2	≥3	
	n = 4,994	n = 2,649	n = 999	n = 438	
**Total**	**55.7 (53.9–57.4)**	**28.7 (27.4–30.2)**	**10.8 (9.9–11.8)**	**4.8 (4.2–5.5)**	—
**Sex**					0.001
Male	57.4 (54.5–60.2)	29.6 (27.4–31.9)	9.5 (8.3–10.9)^§^	3.4 (2.8–4.3)^§^	—
Female	53.8 (51.3–56.4)	28.0 (25.9–30.1)	12.1 (10.6–13.8)	6.1 (5.2–7.2)	—
**Race/Ethnicity^¶^**					0.100
White**	55.0 (52.4–57.5)	28.5 (26.6–30.4)^§§^	11.9 (10.6–13.3)^††^	4.7 (3.9–5.6)	—
Black**	52.2 (48.3–56.1)^††^	32.8 (29.3–36.4)	10.5 (8.3–13.2)	4.5 (3.0–6.9)	—
Hispanic	57.1 (55.4–58.9)	29.0 (26.7–31.5)	9.4 (7.9–11.2)	4.4 (3.3–5.8)	—
**Grade**					0.005
9	53.1 (50.2–56.0)	30.7 (28.1–33.4)	12.4 (10.5–14.6)	3.9 (2.9–5.1)	—
10	53.4 (50.3–56.5)	29.9 (27.6–32.3)	11.4 (9.8–13.3)	5.3 (4.1–6.9)	—
11	57.3 (54.4–60.2)^¶¶,^***	27.7 (25.2,30.3)	10.5 (8.9,12.5)	4.5 (3.5,5.9)	—
12	59.4 (56.1,62.7)^¶¶,^***	26.6 (23.8–29.7)	8.6 (6.7–10.9)^¶¶,^***	5.4 (4.2–6.8)	—
**Sexual identity**					<0.001
Heterosexual	57.8 (56.2–59.4)	28.8 (27.4–30.1)	9.6 (8.7–10.5)	3.8 (3.2–4.6)	—
Gay, lesbian, or bisexual	41.3 (36.5–46.3)^†††^	31.3 (26.8–36.3)	17.8 (14.3–21.9)^†††^	9.6 (7.3–12.4)^†††^	—
Not sure	54.8 (46.2–63.1)^§§§^	21.6 (15.9–28.6)^†††,§§§^	16.2 (12.2–21.2)^†††^	7.4 (4.9–11.0)^†††^	—

**Abbreviation:** CI = confidence interval.

* Types of violence experiences during the 12 months before the survey: physical fighting or threatened with a weapon (in a physical fight, in a physical fight on school property, threatened or injured with a weapon on school property), physical dating violence, sexual violence (sexual violence by anyone, sexual dating violence), and bullying (bullied at school or bullied electronically).

^†^ Test for trend.

^§^ Significantly different from females based on pairwise t-test p<0.05.

^¶^ Race/ethnicity “other” not presented because of limited interpretability.

** Non-Hispanic.

^††^ Significantly different from Hispanic based on pairwise t-test p<0.05.

^§§^ Significantly different from Black based on pairwise t-test p<0.05.

^¶¶^ Significantly different from grade 9 based on pairwise t-test p<0.05.

*** Significantly different from grade 10 based on pairwise t-test p<0.05.

^†††^ Significantly different from heterosexual based on pairwise t-test p<0.05.

^§§§^ Significantly different from gay, lesbian, or bisexual based on pairwise t-test p<0.05.

Experiencing violence was significantly associated with all examined health risk behaviors and conditions (Table 2). Students experiencing three or more types of violence reported high prevalences of health risks: 34.1% missed school because of safety concerns, 33.8% had low academic grades, 13.4% carried a weapon on school property, 18.9% carried a gun, 30.1%–65.4% used substances, 21%–63.4% engaged in risky sexual behavior, 39.1% were overweight or had obesity, 71.4% reported suicidal thoughts or behaviors, and 78.4% felt sad or hopeless.

**TABLE 2:** Prevalence of health risk behaviors and conditions among high school students, by number of types of violence experiences — Youth Risk Behavior Survey, United States, 2019

Characteristic	No. of types of violence experiences* % (95% CI)				P-value^†^
	0	1	2	≥3	
	n = 4,994	n = 2,649	n = 999	n = 438	
**Missed school and low academic grades**					
Missed school because of safety concerns^§^	3.8 (2.8–5.3)	8.3 (6.5–10.6)	18.3 (15.5–21.6)	34.1 (29.2–39.4)	<0.001
Earned mostly Cs/Ds/Fs^¶^	16.2 (13.8–19.0)	24.5 (21.1–28.3)	29.2 (24.3–34.7)	33.8 (27.4–40.9)	<0.001
**Health risk behaviors**					
Weapon carrying					
Carried a weapon on school property^§^	0.9 (0.5–1.5)	3.0 (2.1–4.2)	4.5 (2.9–6.8)	13.4 (9.8–18.1)	<0.001
Carried a gun**	1.5 (0.9–2.4)	5.5 (4.4–7.0)	6.8 (5.0–9.1)	18.9 (14.7–23.9)	<0.001
**Substance use**					
Smoked cigarettes or cigars or used smokeless tobacco^§^	5.2 (4.2–6.6)	12.5 (10.1–15.4)	15.4 (12.3–19.0)	37.0 (30.1–44.4)	<0.001
Used electronic vapor products^§^	23.8 (21.4–26.3)	38.7 (35.7–41.7)	52.2 (48.5–55.8)	65.4 (58.8–71.4)	<0.001
Drank alcohol^§^	21.4 (19.3–23.7)	34.8 (31.8–37.9)	47.7 (42.8–52.5)	59.3 (52.6–65.7)	<0.001
Binge drinking^††^	9.5 (7.9–11.2)	16.4 (14.1–19.0)	20.4 (16.5–24.9)	36.5 (29.0–44.7)	<0.001
Used marijuana^§§^	14.6 (12.8–16.5)	28.1 (25.5–30.8)	31.2 (27.7–35.0)	46.7 (39.6–53.9)	<0.001
Prescription pain medicine misuse^§§^	3.4 (2.7–4.3)	7.1 (5.9–8.7)	12.2 (10.2–14.4)	30.1 (24.1–37.0)	<0.001
**Risky sexual behavior**					
Drank alcohol or used drugs before last sexual intercourse^¶¶^	15.0 (11.6–19.1)	20.2 (16.9–24.0)	27.0 (20.8–34.2)	43.1 (34.9–51.8)	<0.001
Currently sexually active with multiple persons***	2.7 (2.0–3.7)	8.0 (6.3–10.0)	9.3 (6.8–12.5)	21.0 (15.5–27.8)	<0.001
Did not use a condom during last sexual intercourse^¶¶,†††^	39.6 (35.7–43.7)	45.1 (41.7–48.5)	46.6 (38.5–54.9)	63.4 (54.9–71.1)	0.004
**Weight**					
Overweight or obesity^§§§^	29.5 (25.7–33.5)	32.5 (29.3–35.9)	35.5 (30.4–40.9)	39.1 (33.6–44.9)	0.001
**Mental health and suicide risks**					
Felt sad or hopeless^¶¶¶^	24.8 (22.8–27.0)	42.6 (39.6–45.7)	64.2 (59.7–68.4)	78.4 (73.2–82.8)	<0.001
Suicidal thoughts or behavior****	13.7 (12.1–15.5)	27.9 (25.3–30.7)	48.3 (44.9–51.7)	71.4 (64.9–77.2)	<0.001

**Abbreviation:** CI = confidence interval.

* Types of violence experiences during the 12 months before the survey: physical fighting or threatened with weapon (in a physical fight, in a physical fight on school property, threatened or injured with a weapon on school property), physical dating violence, sexual violence (sexual violence by anyone, sexual dating violence), and bullying (bullied at school or bullied electronically).

^†^ Test for trend.

^§^ On at least 1 day during the 30 days before the survey.

^¶^ During the 12 months before the survey.

** On at least 1 day during the 12 months before the survey.

^††^ Had four or more drinks of alcohol in a row (if female) or five or more drinks of alcohol in a row (if male) within a couple of hours on at least 1 day during the 30 days before the survey.

^§§^ One or more times during the 30 days before the survey.

^¶¶^ Among students who were sexually active with one or more persons during the 3 months before the survey

*** Had sex with two or more persons during the 3 months before the survey.

^†††^ Excludes female students who reported sexual contact with only females.

^§§§^ Were ≥85th percentile for body mass index, based on sex- and age-specific reference data from the 2000 CDC growth charts.

^¶¶¶^ Almost every day for ≥2 weeks in a row so that the student stopped doing some usual activities, during the 12 months before the survey.

**** Created by combining affirmative responses to any of following suicide related experiences during the 12 months before the survey: seriously considered attempting suicide, made a plan to attempt suicide, or attempted suicide.

Except for overweight or obesity, the aPR for each examined health risk was significantly higher among youths who experienced one type of violence (range = 1.2–3.9) than those experiencing no types (Table 3). Among students who experienced two types of violence, aPRs for 15 of the outcomes were significantly higher (range = 1.2–6.7) than among those experiencing no types. All 16 outcomes were significantly more prevalent among youths who experienced three or more types (range = 1.3–20.6) than among those who experienced no types. The prevalence of the following health risk behaviors increased in a stepwise manner as the number of violence types increased: missing school because of safety concerns, using electronic vapor products, drinking alcohol, misusing prescription pain medicine, feeling sad or hopeless, and having suicidal thoughts or behavior. Prevalence of the following risk behaviors was higher among students who experienced three or more types of violence than it was among those who experienced two types of violence: carrying a weapon on school property, carrying a gun, using tobacco, engaging in binge drinking, using marijuana, and engaging in all three measures of risky sexual behavior.

**TABLE 3:** Adjusted prevalence ratios for health risk behaviors and conditions among high school students, by number of violence experiences — Youth Risk Behavior Survey, United States, 2019

Characteristic	No. of types of violence experiences*^,† ^aPR (95% CI)		
	1	2	≥3
**Missed school and low academic grades**			
Missed school because of safety concerns^§^	2.1 (1.7–2.6)^¶^	4.6 (3.3–6.4)^¶,^**	8.5 (5.8–12.5)^¶,^**^,††^
Earned mostly Cs/Ds/Fs^§§^	1.5 (1.3–1.7)^¶^	1.8 (1.5–2.2)^¶,^**	2.2 (1.7–2.7)^¶,^**
**Health risk behaviors**			
Weapon carrying			
Carried a weapon on school property^§^	3.9 (2.4–6.2)^¶^	6.7 (3.7–12.0)^¶^	20.6 (11.6–36.6)^¶,^**^,††^
Carried a gun^¶¶^	3.9 (2.4–6.3)^¶^	5.6 (3.2–9.8)^¶^	16.2 (9.7–27.3)^¶,^**^,††^
**Substance use**			
Smoked cigarettes cigars or used smokeless tobacco^§^	2.4 (1.9–3.1)^¶^	2.9 (2.2–3.8)^¶^	7.1 (5.3–9.4)^¶,^**^,††^
Used electronic vapor products^§^	1.7 (1.6–1.8)^¶^	2.3 (2.0–2.6)^¶,^**	2.8 (2.4–3.3)^¶,^**^,††^
Drank alcohol^§^	1.7 (1.5–1.8)^¶^	2.3 (2.0–2.6)^¶,^**	2.7 (2.3–3.2)^¶,^**^,††^
Binge drinking***	1.8 (1.5–2.1)^¶^	2.3 (1.7–3.0)^¶^	3.8 (2.9–4.9)^¶,^**^,††^
Used marijuana^†††^	2.0 (1.7–2.2)^¶^	2.2 (1.9–2.5)^¶^	3.2 (2.7–3.7)^¶,^**^,††^
Prescription pain medicine misuse^†††^	2.1 (1.7–2.5)^¶^	3.3 (2.5–4.5)^¶,^**	8.5 (6.1–11.7)^¶,^**^,††^
**Risky sexual behavior**			
Drank alcohol or used drugs before last sexual intercourse^§§§^	1.4 (1.1–1.9)^¶^	1.9 (1.3–2.7)^¶^	3.1 (2.3–4.2)^¶,^**^,††^
Currently sexually active with multiple persons^¶¶¶^	3.0 (2.1–4.4)^¶^	3.9 (2.5–6.1)^¶^	8.4 (5.7–12.4)^¶,^**^,††^
Did not use a condom during last sexual intercourse^§§§,^****	1.2 (1.0–1.3)^¶^	1.2 (1.0–1.4)	1.6 (1.3–1.8)^¶,^**^,††^
**Weight**			
Overweight or obesity^††††^	1.1 (1.0–1.2)	1.2 (1.1–1.4)^¶^	1.3 (1.1–1.6)^¶^
**Mental health and suicide risk**			
Felt sad or hopeless^§§§§^	1.7 (1.5–1.9)^¶^	2.4 (2.2–2.6)^¶,^**	3.0 (2.7–3.3)^¶,^**^,††^
Suicidal thoughts or behavior^¶¶¶¶^	1.9 (1.7–2.2)^¶^	3.0 (2.6–3.5)^¶,^**	4.7 (4.0–5.6)^¶,^**^,††^

**Abbreviations:** aPR = adjusted prevalence ratio; CI = confidence interval.

* Types of violence experiences during the 12 months before the survey: physical fighting or threatened with weapon (in a physical fight, in a physical fight on school property, threatened or injured with a weapon on school property), physical dating violence, sexual violence (sexual violence by anyone, sexual dating violence), and bullying (bullied at school, bullied electronically).

^†^ Reference for all models is zero types of violence.

^§^ On at least 1 day during the 30 days before the survey.

^¶^ Significantly different from zero types, based on linear contrast analysis (p<0.05).

** Significantly different from one type, based on linear contrast analysis (p<0.05).

^††^ Significantly different from two types, based on linear contrast analysis (p<0.05).

^§§^ During the 12 months before the survey.

^¶¶^ On at least 1 day during the 12 months before the survey.

*** Had four or more drinks of alcohol in a row (if female) or five or more drinks of alcohol in a row (if male) within a couple of hours on at least 1 day during the 30 days before the survey.

^†††^ One or more times during the 30 days before the survey.

^§§§^ Among students who were sexually active with one or more persons during the 3 months before the survey.

^¶¶¶^ Had sex with two or more persons during the 3 months before the survey.

**** Excludes female students who reported sexual contact with only females.

^††††^ Were ≥85th percentile for body mass index, based on sex- and age-specific reference data from the 2000 CDC growth charts.

^§§§§^ Almost every day for ≥2 weeks in a row so that the student stopped doing some usual activities, during the 12 months before the survey.

^¶¶¶¶^ Created by combining affirmative responses to any of following suicide-related experiences in the 12 months before the survey: seriously considered attempting suicide, made a plan to attempt suicide, or attempted suicide.

## Discussion

Experiencing violence was common among U.S. high school students. Approximately one half of high school students experienced at least one type of violence, and approximately one in seven experienced two or more types. Results are consistent with previous research indicating that adverse experiences in childhood are common (*3*,*10*). During the critical developmental period of adolescence, violence that occurs between peers in school and the community was associated with all the health risk behaviors and conditions examined, which could have a negative impact on immediate and long-term health and social outcomes. As adolescents’ experiences with multiple types of violence increased, their likelihood of engaging in risk behaviors increased significantly, often in a stepwise manner. Relative to youths with no reported violence type experiences, adjusted models demonstrated prevalence estimates up to seven times higher among those with two types of violence experiences and up to 21 times higher among those experiencing three or more types of violence.

Some adolescent health behaviors (e.g., carrying a weapon and suicidal behavior) pose an immediate risk for mortality in adolescence when homicide and suicide are among the top three leading causes of death (*1*). Others, such as missing school, low academic grades, and poor mental health, could have long-term implications for high school graduation, well-being, and life opportunities (e.g., employment and income) (*11*). Substance use and sexual risk-taking behaviors in adolescence could have immediate and long-term health implications (e.g., overdose and development of substance use disorder, impaired driving, sexually transmitted infections including human immunodeficiency virus infection, and chronic diseases).

The impact of violence experiences on adolescent brain and interpersonal skill development could result in the adoption of negative coping behaviors (e.g., substance use and unhealthy eating) to handle stress that could harm short- and long-term health (*4*,*6*). These youths might have less developed abilities to problem-solve and manage social interactions in ways that minimize risk (e.g., negotiating safer sex practices and resolving conflicts without violence). Youths who experience violence might have heightened fear and perceived vulnerability, which might contribute to carrying a weapon and missing school. Prior research demonstrates that youth weapon carrying is positively associated with victimization, and many youths who carry weapons report doing so for self-protection (*12*). Violence victimization is also related to suicidal thoughts or behavior and risk-taking behavior (*13*,*14*). Primary prevention approaches (e.g., social-emotional learning, mentoring and after-school programs, and parent and family relationship programs) can build youths’ emotional regulation and communication skills to resolve conflicts without violence and reduce risk behaviors, such as smoking, substance use, weapon carrying, sexual risk taking, and academic challenges (*15*).

This study extends research on health impacts from experiences of violence outside the home but does not include all types of violence that can negatively affect youth development and health. Other forms of community and school violence (e.g., gang-related violence, homophobic name calling, and witnessing violence) are associated with poor health outcomes and well-being (*5*,*16*,*17*). Some youths (e.g., females, racial/ethnic minorities, and sexual minorities) are more likely to experience multiple forms of violence (*4**,**15*). Community factors (e.g., poverty, limited access to high-quality education, unstable housing, bias, and stigma) can contribute to increased risk for violence and other health problems (e.g., coronavirus disease 2019 [COVID-19]) and the differences observed across population subgroups (*18*).* Emerging data suggest that certain forms of violence, including relationship and community violence, might be increasing and that youths might be more vulnerable to online violence (e.g., threats and harassment through social media) during the COVID-19 pandemic (*19*). The influence of community and contextual factors underscores the importance of prevention approaches that build youths’ skills, support families, promote social norms that protect against violence and adversity (e.g., positive norms about gender and parenting), and create protective environments (e.g., positive school climate) (*10*,*15*,*20*).

The findings in this report are subject to at least six limitations. First, recall and social desirability biases might reduce self-reporting of violence experiences and health risk behaviors, thereby underestimating the actual prevalence of these experiences and behaviors. Second, causality and directionality cannot be inferred from these cross-sectional data. Third, some students were not asked all violence questions, and the analytic sample was restricted to students for whom data were available to determine the presence of the four violence types examined. This might reduce generalizability of the results. Fourth, data are student reports, and results might not be generalized to out-of-school youth. Fifth, data were not available to control for other factors (e.g., socioeconomic status of students) that could affect violence experiences and examined health outcomes. Finally, assessed violence experiences focused on those in the 12 months before the survey. Some early and unmeasured experiences of violence (e.g., childhood physical and sexual abuse) that have significant negative impacts on development were not examined, resulting in underestimates of youths’ experiences of violence.

Violence that has negative impacts on healthy development of youths can be prevented (*4**,**15**,**20*). Early intervention is strategic because it is cost-effective and can potentially reduce injury and illness throughout life (*6*,*15*). Strategies for the primary prevention of violence, such as those identified by CDC’s violence prevention technical packages, can put youths on a healthy trajectory by reducing the leading causes of morbidity and mortality among adolescents and adults (*15*). Many strategies can simultaneously reduce more than one type of violence, health disparities, and associated health risk behaviors and conditions (*15*,*20*). These strategies promote adolescents’ short- and long-term health and opportunity by addressing individual behaviors as well as providing quality education, access to supportive services, connections with caring adults, and safe and supportive home, school, and community environments. Building school and community capacity to implement effective violence prevention strategies that reach all youths can promote health across the lifespan. Collaboration of multiple sectors (e.g., public health, public safety, and education) can ensure the effective implementation of strategies to help youths and communities be safe and thrive.

**Summary**

**What is already known about this topic?**

Violence during adolescence is a leading cause of death. Violence can harm development and affects health across the lifespan.

**What is added by this report?**

Adolescents experience multiple forms of violence at school and in the community. Experiencing violence was significantly associated with higher prevalence of all examined health risks and conditions, including missing school, low academic grades, health risk behaviors, and mental health and suicide risks.

**What are the implications for public health practice?**

Violence is preventable. Primary prevention puts adolescents on a healthy developmental trajectory by reducing the leading causes of morbidity and mortality among youths and young adults.

## Footnotes

*
https://www.cdc.gov/coronavirus/2019-ncov/community/health-equity/race-ethnicity.html
